# Effects of astaxanthin‐rich dried cell powder from *Paracoccus carotinifaciens* on carotenoid composition and lipid peroxidation in skeletal muscle of broiler chickens under thermo‐neutral or realistic high temperature conditions

**DOI:** 10.1111/asj.13141

**Published:** 2018-12-16

**Authors:** Hiroki Inoue, Saki Shimamoto, Hironori Takahashi, Yuki Kawashima, Sato Wataru, Daichi Ijiri, Akira Ohtsuka

**Affiliations:** ^1^ Department of Biochemical Science and Technology Kagoshima University Kagoshima Japan; ^2^ The United Graduate School of Agricultural Sciences Kagoshima University Kagoshima Japan; ^3^ Biotechnology Business Group Biotechnology Business Unit High Performance Materials Company JXTG Nippon Oil Energy Corporation Tokyo Japan; ^4^ Biotechnology Development Group Biotechnology Business Unit High Performance Materials Company JXTG Nippon Oil Energy Corporation Yokohama Japan; ^5^ Present address: Business Promotion Group V. HPM Business Promotion Department High Performance Materials Company JXTG Nippon Oil Energy Corporation Tokyo Japan; ^6^ Present address: ICC/CT Sales & Marketing Group Sales & Marketing Department 2 IS JAPAN CO., LTD. Saitama Japan

**Keywords:** antioxidant, carotenoid, chicken, heat stress, lipid peroxidation

## Abstract

Thirty‐two 15‐day old broiler chicks (Chunky strain ROSS 308) were randomly divided into four treatments in a 2 × 2 factorial design. The main factors were diet (basal diet or basal diet supplemented with 0.15% astaxanthin‐rich dried cell powder (Panaferd‐P [astaxanthin 30 ppm]) and ambient temperature (thermo‐neutral [25 ± 1°C] or high [35 ± 1°C for 6 hr]). Dietary supplementation with Panaferd‐P did not affect growth performance, though high ambient temperature decreased feed intake and the weight of breast tender muscle, liver, and heart. High ambient temperature also decreased redness in both breast and leg muscles of chickens, while Panaferd‐P increased redness and yellowness of breast and leg muscles of chickens. Panaferd‐P increased *Paracoccus carotinifaciens*‐derived pigments (i.e., adonixanthin, astaxanthin, adonirubin, and cantaxanthin) as well as corn‐derived pigments such as zeaxanthin and lutein in breast and leg muscles. High ambient temperature increased the malondialdehyde (MDA) concentration in breast muscle, while Panaferd‐P decreased the MDA concentration in breast muscle under both temperature conditions. Our results suggest that dietary supplementation with Panaferd‐P increases muscle carotenoid content, the redness and yellowness of meat and decreases the muscle MDA concentration in broiler chickens kept under thermo‐neutral or high ambient temperature conditions.

## 1 INTRODUCTION

Ambient temperatures above the thermo‐neutral zone cause environmental heat stress. Chickens are more vulnerable to heat stress than other domestic animals because they lack sweat glands and have higher body temperatures (Ensminger, Oldfield, & Heineman, 1990; Sahin, Sahin, Kucuk, Hayirili, & Prasad, 2009). Heat is thus a major stressor for chickens, resulting in a range of physiological alterations such as severely depressed growth performance, carcass yield and meat quality, and red coloration of meat (Whitehead & Keller, 2003; Zeferino et al., 2016).

Reactive oxygen species (ROS), such as singlet oxygen, hydrogen peroxide, and hydroxyl radicals, are highly reactive molecules produced in mitochondria (Freeman & Crapo, 1982; Khan & Wilson, 1995). Under physiological conditions, ROS have functional roles as antimicrobial agents and cellular signaling molecules, although they should be neutralized by antioxidant system due to their harmful actions for homeostasis (Surai, 2002; Valko et al., 2007). Under high ambient temperature conditions, ROS generation increases in various body tissues as the heat load elevates (Khan et al., 2012). The increased ROS content oxidizes and impairs lipids, proteins, and DNA (Liu, Wen, Liu, & Li, 1999). Moreover, chicken muscle contains a high polyunsaturated fatty acid content, making it susceptible to oxidative deterioration (Igene & Pearson, 1979; Wilson, Pearson, & Shorland, 1976).

Numerous studies have demonstrated that antioxidant vitamins (e.g., vitamins C and E) have direct or indirect roles in deactivating ROS (Seifried, Anderson, Fisher, & Milner, 2007); however, their potency decreases under high ambient temperature conditions (McDowell, 1989; Shimizu et al., 2006; Surai, 2002). Several methods have been established to alleviate the ROS production and/or lipid oxidation induced by high ambient temperatures in the skeletal muscle of chickens. For instance, nutritional supplementation with the aforementioned vitamins has proven beneficial to minimize the adverse effects of heat stress in chickens (Eid, Ebeid, Moawad, & El‐Habbak, 2008; Lin, Jiao, Buyse, & Decuyperre, 2006).

Astaxanthin, a red carotenoid, has the antioxidant role of quenching singlet oxygen (Kamezaki et al., 2016; Kurashige, Okimasu, Inoue, & Utsumi, 1990; Naguib, 2000). Its antioxidant activity is approximately 10 times higher than that of other carotenoids (e.g., zeaxanthin, lutein, tunaxanthin, cantaxanthin, and β‐carotene) and 100 times greater than vitamin E (α‐tocopherol) (Miki, 1991). In addition to its antioxidant activity, astaxanthin has been used as pigment, e.g., in salmonid and crustacean aquaculture, dietary supplementation with astaxanthin is applied to generate the pink color (Higuera‐Ciapara, Félix‐Valenzuela, & Goycoolea, 2006). Likewise, in chickens, dietary supplementation with astaxanthin deriving from *Phaffia rhodozyma* increased the redness of muscle and egg yolk (Akiba, Sato, Takahashi, Matsushita, et al., 2001; Akiba, Sato, Takahashi, Takahashi, et al., 2000; Akiba, Sato, Takahashi, Toyomizu, et al., 2000; Akiba, Sato, Takahashi, Toyomizu, et al., 2001).

The aim of this study was to examine the effects of dietary supplementation with astaxanthin on color, carotenoid composition, and lipid peroxidation levels of the skeletal muscle of broiler chickens kept under thermo‐neutral or realistic high ambient temperature conditions. As dietary supplement, an astaxanthin‐rich dried cell powder (Panaferd‐P) from carotenoid‐producing bacteria (*Paracoccus carotinifaciens*) was used.

## 2 MATERIALS AND METHODS

### 2.1 Animals and experimental design

All experimental protocols and procedures were reviewed and approved by the Animal Care and Use Committee of Kagoshima University. One hundred 1‐day‐old male broiler chicks (Chunky strain ROSS 308) were obtained from a commercial hatchery (Kumiai Hina Center, Kagoshima, Japan). Chicks were housed in an electrically heated battery brooder and provided with water and a commercial diet (23% crude protein, 12.8 MJ/kg; Nichiwa Sangyou Company, Hyogo, Japan) until they were 12 days old. On Day 12, 32 chicks were randomly selected from the group of 100. These chicks were housed individually in wire‐bottomed aluminum cages (50 × 40 × 60 cm) and fed a basal diet (see Table 1 for diet composition) for 3 days until beginning of the main experimental period. Heat exposure experiment was designed according to our previous studies (El‐Deep, Ijiri, Ebeid, & Ohtsuka, 2016; El‐Deep, Ijiri, Eid, Yamanaka, & Ohtsuka, 2014). Panaferd‐P supplementation (0.15%) was determined to adjust to the Astaxanthin concentration (30 ppm) of the previous study (Akiba, Sato, Takahashi, Matsushita, et al., 2001). Chicks were then randomly allocated to one of four groups (2 × 2 factorial design), where the main factors were diet (basal diet or basal diet supplemented with 0.15% Panaferd‐P [astaxanthin 30 ppm]) and ambient temperature (thermo‐neutral temperature of 25 ± 1°C or high ambient temperature of 35 ± 1°C). The experiment was conducted in a temperature‐controlled room with 24 hr of light and 50%–70% relative humidity. Chicks assigned to the high ambient temperature challenge were kept at 35 ± 1°C for 6 hr every day to mimic realistic summer conditions. At 28 days old, all chickens were weighed, anesthetized with carbon dioxide and killed by cervical dislocation. Chickens were dissected and the weights of breast muscle (*pectoralis major muscle*), breast tender muscle (*pectoralis minor muscle*), leg muscles (thigh and drumstick), liver, heart, and abdominal fat tissue were measured. Blood samples were collected in heparinized test tubes, centrifuged at 5,900 × *g* for 10 min at 4°C to separate plasma, and stored at −30°C until analysis. A portion of the breast muscle was snap frozen in liquid nitrogen and stored at −80°C until use for the measurement of thiobarbituric acid reactive substances (TBARS), vitamin E (α‐tocopherol), and carotenoids (lutein, zeaxanthin, canthaxanthin, adonirubin, astaxanthin, and adonixanthin).

**Table 1:** Composition and analysis of the basal diet

	%
Ingredients (g/100 g)	
Corn meal	57.90
Soybean meal	34.00
Corn oil	4.30
CaHPO_4_	2.00
CaCO_3_	0.66
NaCl	0.50
DL‐Methionine	0.14
Mineral and vitamin premix^a^	0.50
Calculated analysis	
Crude protein (%)	20.00
Metabolizable energy (MJ/kg)	3.10

^a^Content per kg of the vitamin and mineral premix: vitamin A 90 mg, vitamin D3 1 mg, DL‐alpha‐tocopherol acetate 2,000 mg, vitamin K3 229 mg, thiamin nitrate 444 mg, riboflavin 720 mg, calcium d‐pantothenate 2,174 mg, nicotinamide 7,000 mg, pyridoxine hydrochloride 700 mg, biotin 30 mg, folic acid 110 mg, cyanocobalamine 2 mg, calcium iodate 108 mg, MgO 198,991 mg, MnSO_4_ 32,985 mg, ZnSO_4_ 19,753 mg, FeSO_4_ 43,523 mg, CuSO_4_ 4,019 mg and choline chloride 299,608 mg.

### 2.2 Meat color analysis

The color of breast and leg muscles was determined using a colorimeter (Konica Minolta, CR‐400, Tokyo, Japan) using the following parameters: L* (lightness), a* (redness) and b* (yellowness).

### 2.3 Determination of malondialdehyde concentration

To evaluate lipid peroxidation levels in skeletal muscle, the malondialdehyde (MDA) concentration in breast muscle was determined colorimetrically as TBARS according to the method described by Azada, Kikusato, Maekawa, Shirakawa, and Toyomizu (2010). In brief, 300 mg of breast muscle was homogenized in 1 ml of 154 mM KCl and centrifuged at 700 × *g*, and the supernatant was collected. Forty microliters of the supernatant was mixed with 40 μl of 8.1% sodium dodecyl sulfate, 300 μl of 20% acetate buffer (pH 3.5), and 300 μl of 0.8% 2‐thiobarbituric acid. After vortexing, the sample was incubated at 95°C for 60 min and then transferred to ice. After addition of 1 ml of butanol‐pyridine (15:1 v/v), the sample was mixed by vortexing and centrifuged at 1,200 × *g* for 10 min. Absorbance of the supernatant was measured at 532 nm. The TBARS content was expressed as the equivalent level of MDA.

### 2.4 Determination of α‐tocopherol concentration

One hundred milligrams of breast muscle was homogenized in 1 ml of 10 mM Tris, 150 mM NaCl, and 1 mM EDTA‐2Na (pH 7.4). Five hundred microliters of the homogenate was mixed with 1 ml of hexan‐2 propanol (6:4 v/v), and centrifuged at 20,000 × *g* for 3 min. The supernatant was evaporated and reconstituted in 500 μl of ethanol with 0.025% butylated hydroxytoluene. The α‐tocopherol concentration in breast muscle was determined using the LC‐2000 Plus HPLC System (JASCO Co. Ltd, Tokyo, Japan) with an Inertsil ODS‐3 Column (4.6 × 250 mm; GL Science Inc., Tokyo, Japan) according to the method described by Faustman et al. (1989).

### 2.5 Determination of carotenoid concentration

Determination of carotenoid concentration in breast muscle, leg muscles, and abdominal fat tissue was outsourced to the Research Institute for Production Development (Kyoto, Japan).

### 2.6 Statistical analysis

Data are expressed as means ± *SEM*. Comparisons were performed using Tukey's multiple comparison test. *p* Values under 5% were considered statistically significant. All analyses were performed using the general linear model procedure of the statistical analysis system software package (SAS/STAT Version 9.3; Statistical Analysis Systems Institute Inc., Cary, NC).

## 3 RESULTS AND DISCUSSION

Final body weight, body weight gain, and feed conversion ratio were not affected by ambient temperature or Panaferd‐P dietary supplementation, while feed intake was significantly depressed in chickens kept under high ambient temperature (Table 2). In addition, the body temperature of chickens kept under high ambient temperature was significantly increased compared with that of chickens kept under thermo‐neutral conditions. Although the weight of breast muscle and abdominal fat tissue were not affected by temperature, the weight of breast tender muscle, liver and heart were decreased in chickens kept under high ambient temperature (Table 3). These results suggest that the high ambient temperature condition used in this study (35°C for 6 hr per day) could induce the negative effects commonly observed in broiler chickens under heat stress, i.e., depression of feed intake, increase in body temperature, and decrease in the weight of breast tender muscle, liver, and heart. Dietary supplementation with Panaferd‐P failed to alleviate these heat‐induced negative effects.

**Table 2:** Effects of dietary supplementation with Panaferd‐P on growth performance parameters of broiler chickens kept under thermo‐neutral or high ambient temperature conditions

	Thermo‐neutral temperature (25 ± 1°C)		Heat ambient temperature (35 ± 1°C for 6 hr/day)		Panaferd‐P	Temperature	*P* × *T*
	Control	Panaferd‐P	Control	Panaferd‐P			
Final body weight	1,098.00 ± 49.63	1,069.76 ± 73.95	1,020.74 ± 25.63	990.84 ± 26.27	0.4267	0.2437	0.7392
Body weight gain	716.19 ± 46.46	678.44 ± 69.28	636.80 ± 22.40	602.44 ± 23.69	0.4126	0.1971	0.6892
Feed intake	1,059.02 ± 64.94	1,093.78 ± 90.06	988.06 ± 25.57	922.04 ± 50.37	0.7904	0.0465	0.3945
Feed conversion ratio	1.59 ± 0.07	1.70 ± 0.12	1.55 ± 0.03	1.58 ± 0.03	0.3474	0.2553	0.5707
Body temperature	40.58 ± 0.16^b^	40.65 ± 0.18^b^	42.55 ± 0.24^a^	42.74 ± 0.49^a^	0.6157	<0.0001	0.8293

*Note*

Results are expressed as *M* ± *SEM* (*n* = 8). Means with the same superscript letter within rows are not significantly different at *p *<* *0.05. Panaferd‐P, the effect of Panaferd‐P; Temperature, the effect of high ambient temperature; *P* × *T*, the statistical interaction between Panaferd‐P and high ambient temperature.

**Table 3:** Effects of dietary supplementation with Panaferd‐P on tissue weights of broiler chickens kept under thermo‐neutral or high ambient temperature conditions

	Thermo‐neutral temperature (25 ± 1°C)		Heat ambient temperature (35 ± 1°C for 6 hr/day)		Panaferd‐P	Temperature	*P* × *T*
	Control	Panaferd‐P	Control	Panaferd‐P			
Breast muscle	176.63 ± 12.05	177.02 ± 14.22	164.33 ± 4.95	157.13 ± 8.24	0.7110	0.0872	0.6792
Breast tender muscle	40.01 ± 2.20	40.21 ± 2.95	36.32 ± 1.57	34.70 ± 1.52	0.7066	0.0193	0.6283
Leg muscles	192.26 ± 11.85	185.62 ± 13.84	192.85 ± 5.16	185.31 ± 8.09	0.9153	0.0017	0.8869
Liver	19.02 ± 1.25	19.26 ± 1.39	15.81 ± 0.64	15.78 ± 1.04	0.6432	<0.0001	0.6107
Heart	4.66 ± 0.51^a^	4.68 ± 0.53^a^	3.33 ± 0.10^b^	3.01 ± 0.09^b^	0.6716	0.0378	0.4186
Abdominal fat tissue	7.97 ± 1.32	8.44 ± 1.75	11.57 ± 1.04	10.07 ± 1.33	0.4335	0.9875	0.9601

*Note*

Results are expressed as *M* ± *SEM* (*n* = 8). Means with the same superscript letter within rows are not significantly different at *p *<* *0.05. Panaferd‐P, the effect of Panaferd‐P; Temperature, the effect of high ambient temperature; *P* ×* T*, the statistical interaction between Panaferd‐P and high ambient temperature.

It has been reported that heat stress can affect meat color, e.g., redness is notably decreased in the breast muscle of chickens kept under high ambient temperature conditions (Zeferino et al., 2016; Zhang et al., 2012). In agreement with the previous literature, the redness of both the breast and leg muscles in chickens kept under high ambient temperature was decreased (tendency for breast muscle, *p *=* *0.07; significant difference for leg muscles, *p *<* *0.05) compared with those of chickens kept under thermo‐neutral conditions in the present study (Table 4). This decrease in muscle redness might be a result of an alteration in muscle myoglobin concentration, because this is the main protein responsible for meat color (Mancini & Hunt, 2005). However, Zeferino et al. (2016) found that feed restriction could also result in a decrease in muscle redness in broiler chickens. Likewise, in the present study, feed intake was decreased in chickens kept under high ambient temperature (Table 4), suggesting that muscle color in broiler chickens kept at these temperatures might be changed as a result of depression of feed intake.

**Table 4:** Effects of dietary supplementation with Panaferd‐P on the color of breast and leg muscles in broiler chickens kept under thermo‐neutral or high ambient temperature conditions

		Thermo‐neutral temperature (25 ± 1°C)		Heat ambient temperature (35 ± 1°C for 6 hr/day)		Panaferd‐P	Temperature	*P* × *T*
		Control	Panaferd‐P	Control	Panaferd‐P			
Breast muscle	L*	53.30 ± 0.77	51.51 ± 0.85	53.07 ± 0.86	51.43 ± 0.73	0.0311	0.8418	0.9169
	a*	3.44 ± 0.34^ab^	4.50 ± 0.40^a^	2.60 ± 0.39^b^	3.99 ± 0.35^ab^	0.0014	0.0648	0.6393
	b*	8.40 ± 0.32^b^	15.01 ± 0.63^a^	8.03 ± 0.40^b^	13.62 ± 0.27^a^	<0.0001	0.0354	0.2136
Leg muscles	L*	50.62 ± 1.02	50.34 ± 0.96	53.39 ± 0.90	50.57 ± 0.91	0.0909	0.1026	0.1631
	a*	4.70 ± 0.23^b^	6.20 ± 0.63^a^	2.47 ± 0.22^c^	4.97 ± 0.33^ab^	<0.0001	<0.0001	0.1824
	b*	9.59 ± 0.64^b^	10.22 ± 0.71^ab^	9.65 ± 0.56^b^	11.97 ± 0.11^a^	0.0084	0.0918	0.1157

*Note*

Results are expressed as *M* ± *SEM* (*n* = 8). Means with the same superscript letter within rows are not significantly different at *p *<* *0.05. Panaferd‐P, the effect of Panaferd‐P; Temperature, the effect of high ambient temperature; *P* × *T*, the statistical interaction between Panaferd‐P and high ambient temperature.

**Figure 1:**
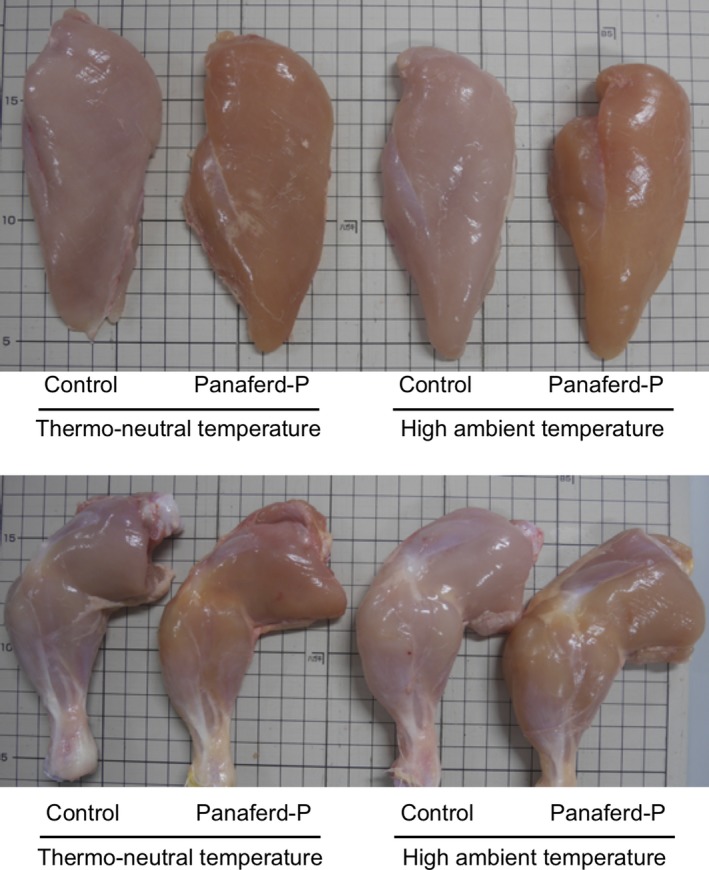
Representative samples of breast and leg muscles of broiler chickens kept under thermo‐neutral and high ambient temperature conditions and fed a basal diet or Panaferd‐P‐supplemented diet

Dietary supplementation with astaxanthin‐containing materials (e.g., *Phaffia rhodozyma* dried cell powder) has also been shown to increase muscle redness in broiler chickens (Akiba, Sato, Takahashi, Matsushita, et al., 2001; Akiba, Sato, Takahashi, Toyomizu, et al., 2001). In agreement with these studies, dietary supplementation with Panaferd‐P markedly changed the muscle color in broiler chickens (Figure 1), increasing the redness of both breast and leg muscles (Table 4). Consequently, although redness of breast and leg muscles was decreased in chickens kept under high ambient temperature, the redness of these muscles was not decreased when chickens were fed the Panaferd‐P‐containing diet. These results suggest that dietary supplementation with Panaferd‐P might ameliorate the high ambient temperature‐induced decrease in muscle redness in broiler chickens.

In addition to redness, feeding the Panaferd‐P‐containing diet also increased the yellowness of breast and leg muscles in broiler chickens (Table 4). Panaferd‐P contains around 4% carotenoids, predominantly astaxanthin (2.2%), adonirubin (1.3%), and cantaxanthin (0.4%) (Bories et al., 2007). Since adonixanthin, cantaxanthin, zeaxanthin, and lutein possess an orange and/or yellow color, the increased yellowness in the breast muscle of broiler chickens fed the Panaferd‐P‐supplemented diet might be due to an accumulation of these yellow pigments. We also determined the carotenoid composition of plasma, breast, and leg muscles. Table 5 shows that *Paracoccus carotinifaciens*‐derived pigments (i.e., astaxanthin, adonixanthin, cantaxanthin, and adonirubin) were detected in plasma and breast and leg muscles of chickens fed the Panaferd‐P‐supplemented diet, while these pigments were not detected in chickens fed the basal diet. Interestingly, corn‐derived pigments such as zeaxanthin and lutein were also increased in both the breast and leg muscle of broiler chickens fed the Panaferd‐P‐supplemented diet. However, a previous study showed that dietary supplementation with *Phaffia rhodozyma*‐derived pigments did not increase yellowness in breast, breast tender, or thigh muscle of chickens (Akiba, Sato, Takahashi, Matsushita, et al., 2001; Akiba, Sato, Takahashi, Toyomizu, et al., 2001). Further research is therefore necessary to determine the reason for this discrepancy in the effect of *Paracoccus carotinifaciens*‐ and *Phaffia rhodozyma*‐derived pigments on meat color in broiler chickens.

**Table 5:** Effects of dietary supplementation with Panaferd‐P on carotenoid concentration in plasma, breast muscle, and leg muscles of broiler chickens kept under thermo‐neutral or high ambient temperature conditions

	Thermo‐neutral temperature (25 ± 1°C)		Heat ambient temperature (35 ± 1°C for 6 hr/day)	
	Control	Panaferd‐P	Control	Panaferd‐P
Plasma				
Astaxanthin		791.1		732.6
Adonixanthin		882.0		792.7
Canthaxanthin		633.1		791.1
Adonirubin		1,076.5		951.5
Lutein	723.5	882.0	684.0	803.4
Zeaxanthin	836.5	1,294.4	757.9	1,095.8
Others	462.9	1,150.3	494.6	899.1
Brest muscle				
Astaxanthin		48.8		61.6
Adonixanthin		53.1		44.1
Canthaxanthin		7.3		3.9
Adonirubin		27.5		28.2
Lutein	23.1	58.0	28.6	43.6
Zeaxanthin	26.8	86.6	33.7	67.0
Others	13.9	169.6	19.8	136.3
Leg muscles				
Astaxanthin		47.0		79.0
Adonixanthin		37.0		46.0
Canthaxanthin		9.0		17.0
Adonirubin		30.0		54.0
Lutein	15.0	58.0	30.0	42.0
Zeaxanthin	18.0	31.0	34.0	46.0
Others	11.0	98.0	20.0	90.0

**Figure 2:**
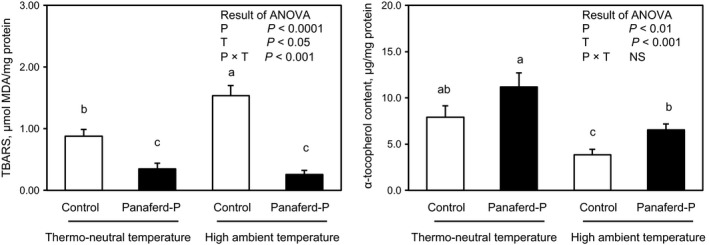
Effects of dietary supplementation with Panaferd‐P on malondialdehyde (MDA) (a) and α‐tocopherol (b) concentrations in breast muscle of broiler chickens kept under thermo‐neutral or high ambient temperature conditions. Results are expressed as *M* ± *SEM* (*n* = 8). Means with the same superscript letter within columns are not significantly different at *p* < 0.05. *P*, the effect of Panaferd‐P; *T*, the effect of high ambient temperature; *P* × *T*, the statistical interaction between Panaferd‐P and high ambient temperature

MDA concentration was increased in the breast muscle of chickens kept under high ambient temperature compared to that of chickens kept under thermo‐neutral conditions (Figure 2a). Dietary supplementation with Panaferd‐P decreased breast muscle MDA concentration under both thermo‐neutral and high ambient temperature conditions. Although vitamin E has an ability to scavenge and remove hydroxy radical and singlet oxygen (Asghar et al., 1991; Faustman et al., 1989; Gray, Gomaa, & Buckley, 1996), the vitamin E concentration in chicken muscle is decreased when chickens are reared under high ambient temperature, and thus a higher MDA concentration is observed in their skeletal muscles (El‐Deep et al., 2014, 2016; Mujahid, Akiba, & Toyomizu, 2009). In agreement with the previous researchers, decreased α‐tocopherol (vitamin E) and increased MDA concentrations were found in the breast muscle of chickens kept under high ambient temperature compared with those of chickens kept under thermo‐neutral conditions in the present study. Importantly, dietary supplementation with Panaferd‐P ameliorated these heat‐induced negative effects on breast muscle. This finding might be partially a result of the antioxidant activity of the carotenoids found in Panaferd‐P (i.e., astaxanthin, adonixanthin, cantaxanthin, and adonirubin). Astaxanthin and other carotenoids have stronger free radical scavenging activity against singlet oxygen than vitamin E (Di Mascio, Devasagayam, Kaiser, & Sies, 1990; Kamezaki et al., 2016). Singlet oxygen reacts with unsaturated fatty acids to produce lipid hydroperoxide, and consequently the level of lipid peroxidation is raised. Since dietary supplementation with Panaferd‐P alleviates both the high ambient temperature‐induced increase in MDA concentration and decrease in vitamin E concentration, Panaferd‐P may be a beneficial feed additive for chickens that reduces the oxidative stress induced by high ambient temperature conditions.

In conclusion, dietary supplementation with Panaferd‐P, an astaxanthin‐rich dried cell powder from *Paracoccus carotinifaciens*, increased both the redness and yellowness of skeletal muscle, and decreased the muscle MDA concentration, by increasing the muscle carotenoid concentration in broiler chickens under both thermo‐neutral and high ambient temperature conditions.

## References

[asj13141-bib-0001] Akiba, Y. , Sato, K. , Takahashi, K. , Matsushita, K. , Komiyama, H. , Tsunekawa, H. , & Nagao, H. (2001). Meat color modification in broiler chickens by feeding yeast *Phaffia rhodozyma* containing high concentrations of astaxanthin. The Journal of Applied Poultry Research, 10, 154–161.

[asj13141-bib-0002] Akiba, Y. , Sato, K. , Takahashi, K. , Takahashi, Y. , Furuki, A. , Kohashi, S. , … Nagao, H. (2000). Pigmentation of egg yolk with yeast *Phaffia rhodozyma* containing high concentration of astaxanthin in laying hens fed on a low‐carotenoid diet. Japanese Poultry Science, 37, 77–85.

[asj13141-bib-0003] Akiba, Y. , Sato, K. , Takahashi, K. , Toyomizu, M. , Matushita, K. , & Komiyama, H. (2001). Meat color of broiler chickens as affected by age and feeding of yeast *Phaffia rhodozyma* containing high concentrations of astaxanthin. Animal Science Journal, 72, 147–153.

[asj13141-bib-0004] Akiba, Y. , Sato, K. , Takahashi, K. , Toyomizu, M. , Takahashi, Y. , Tsunekawa, H. , … Nagao, H. (2000). Availability of cell wall‐fractured yeast, *Phaffia rhodozyma*, containing high concentration of astaxanthin for egg yolk pigmentation. Animal Science Journal, 71, 255–260.

[asj13141-bib-0005] Asghar, A. , Gray, J. I. , Booren, A. M. , Gomaa, E. A. , Abouzied, M. M. , Miller, E. R. , & Buckley, D. J. (1991). Effects of supranutritional dietary vitamin E levels on subcellular deposition of α‐tocopherol in the muscle and pork quality. Journal of the Science of Food and Agriculture, 57, 31–41.

[asj13141-bib-0006] Azada, M. A. K. , Kikusato, M. , Maekawa, T. , Shirakawa, H. , & Toyomizu, M. (2010). Metabolic characteristics and oxidative damage to skeletal muscle in broiler chickens exposed to chronic heat stress. Comparative Biochemistry and Physiology Part A, 155, 401–406. 10.1016/j.cbpa.2009.12.011 20036750

[asj13141-bib-0007] Bories, G. , Brantom, P. , de Barberà, J. B. , Chesson, A. , Cocconcelli, P. S. , Debski, B. , … Wester, P. (2007). Safety and efficacy of Panaferd‐AX (red carotenoid‐rich bacterium *Paracoccus carotinifaciens*) as feed additive for salmon and trout. The EFSA Journal, 546, 1–30.

[asj13141-bib-0008] Di Mascio, P. , Devasagayam, T. P. , Kaiser, S. , & Sies, H. (1990). Carotenoids, tocopherols and thiols as biological singlet molecular oxygen quenchers. Biochemical Society Transactions, 18, 1054–1056. 2088803 10.1042/bst0181054

[asj13141-bib-0009] Eid, Y. , Ebeid, T. , Moawad, M. , & El‐Habbak, M. (2008). Reduction of dexamethasone‐induced oxidative stress and lipid peroxidation in laying hens by dietary vitamin E supplementation. Emirates Journal of Food and Agriculture, 20, 28–40.

[asj13141-bib-0010] El‐Deep, M. H. , Ijiri, D. , Ebeid, T. A. , & Ohtsuka, A. (2016). Effects of dietary nano‐selenium supplementation on growth performance, antioxidative status, and immunity in broiler chickens under thermoneutral and high ambient temperature conditions. The Journal of Poultry Science, 53, 274–283. 10.2141/jpsa.0150133 PMC7477162 32908394

[asj13141-bib-0011] El‐Deep, M. H. , Ijiri, D. , Eid, Y. Z. , Yamanaka, H. , & Ohtsuka, A. (2014). Effects of dietary supplementation with *Aspergillus awamori* on growth performance and antioxidative status of broiler chickens exposed to high ambient temperature. The Journal of Poultry Science, 51, 281–288. 10.2141/jpsa.0150133 PMC7477162 32908394

[asj13141-bib-0012] Ensminger, M. E. , Oldfield, J. E. , & Heineman, W. W. (1990). Feeds and nutrition (2nd ed.). Upper Saddle River, NJ: Prentice Hall.

[asj13141-bib-0013] Faustman, C. , Cassens, R. G. , Schaefer, D. M. , Buege, D. R. , Williams, S. N. , & Scheller, K. K. (1989). Improvement of pigment and lipid stability in Holstein steer beef by dietary supplementation with vitamin E. Journal of Food Science, 54, 858–862.

[asj13141-bib-0014] Freeman, B. A. , & Crapo, J. D. (1982). Biology of disease: Free radicals and tissue injury. Laboratory Investigation, 47, 412–426. 6290784

[asj13141-bib-0015] Gray, J. I. , Gomaa, E. A. , & Buckley, D. J. (1996). Oxidative quality and shelf life of meats. Meat Science, 43, 111–123. 22060645 10.1016/0309-1740(96)00059-9

[asj13141-bib-0016] Higuera‐Ciapara, I. , Félix‐Valenzuela, L. , & Goycoolea, F. M. (2006). Astaxanthin: A review of its chemistry and applications. Critical Reviews in Food Science and Nutrition, 46, 185–196. 16431409 10.1080/10408690590957188

[asj13141-bib-0017] Igene, J. O. , & Pearson, A. M. (1979). Role of phospholipids and triglycerides in warmed‐over flavour development in meat model systems. Journal of Food Science, 44, 1285–1290.

[asj13141-bib-0018] Kamezaki, C. , Nakashima, A. , Yamada, A. , Uenishi, S. , Ishibashi, H. , Shibuya, N. , … Kogure, K. (2016). Synergistic antioxidative effect of astaxanthin and tocotrienol by co‐encapsulated in liposomes. Journal of Clinical Biochemistry and Nutrition, 59, 100–106. 27698536 10.3164/jcbn.15-153 PMC5018571

[asj13141-bib-0019] Khan, R. U. , Naz, S. , Nikousefat, Z. , Selvaggi, M. , Laudadio, V. , & Tufarelli, V. (2012). Effect of ascorbic acid in heat‐stressed poultry. World's Poultry Science Journal, 68, 477–489.

[asj13141-bib-0020] Khan, A. U. , & Wilson, T. (1995). Reactive oxygen species as cellular messengers. Chemistry and Biology, 2, 437–445. 9383446 10.1016/1074-5521(95)90259-7

[asj13141-bib-0021] Kurashige, M. , Okimasu, E. , Inoue, M. , & Utsumi, K. (1990). Inhibition of oxidative injury of biological membranes by astaxanthin. Physiological Chemistry and Physics and Medical NMR, 22, 27–38. 2084711

[asj13141-bib-0022] Lin, H. , Jiao, H. C. , Buyse, J. , & Decuyperre, E. (2006). Strategies for preventing heat stress in poultry. World's Poultry Science Journal, 62, 71–86.

[asj13141-bib-0023] Liu, D. , Wen, J. , Liu, J. , & Li, L. (1999). The roles of free radicals in amyotrophic lateral sclerosis: Reactive oxygen species and elevated oxidation of protein, DNA, and membrane phospholipids. FASEB Journal, 13, 2318–2328. 10593879 10.1096/fasebj.13.15.2318

[asj13141-bib-0024] Mancini, R. A. , & Hunt, M. C. (2005). Current research in meat color. Meat Science, 71, 100–121. 22064056 10.1016/j.meatsci.2005.03.003

[asj13141-bib-0025] McDowell, L. R. (1989). Vitamins in animal nutrition—comparative aspects to human nutrition. Vitamin A and E. London, UK: Academic Press.

[asj13141-bib-0026] Miki, W. (1991). Biological functions and activities of animal carotenoids. Pure and Applied Chemistry, 63, 141–146.

[asj13141-bib-0027] Mujahid, A. , Akiba, Y. , & Toyomizu, M. (2009). Olive oil‐supplemented diet alleviates acute heat stress‐induced mitochondrial ROS production in chicken skeletal muscle. American Journal of Physiology—Regulatory, Integrative and Comparative Physiology, 297, 690–698. 10.1152/ajpregu.90974.2008 19553496

[asj13141-bib-0028] Naguib, Y. M. (2000). Antioxidant activities of astaxanthin and related carotenoids. Journal of Agriculture and Food Chemistry, 48, 1150–1154. 10.1021/jf991106k 10775364

[asj13141-bib-0029] Sahin, K. , Sahin, N. , Kucuk, O. , Hayirili, A. , & Prasad, A. S. (2009). Role of dietary zinc in heat stressed poultry: A review. Poultry Science, 88, 2176–2183. 10.3382/ps.2008-00560 19762873

[asj13141-bib-0030] Seifried, H. E. , Anderson, D. E. , Fisher, E. I. , & Milner, J. A. (2007). A review of the interaction among dietary antioxidants and reactive oxygen species. Journal of Nutritional Biochemistry, 18, 567–579. 17360173 10.1016/j.jnutbio.2006.10.007

[asj13141-bib-0031] Shimizu, N. , Hosogi, N. , Hyon, G. S. , Jiang, S. , Inoue, K. , & Park, P. (2006). Reactive oxygen species (ROS) generation and ROS‐induced lipid peroxidation are associated with plasma membrane modifications in host cells in response to AK‐toxin I from Alternaria alternata Japanese pear pathotype. Journal of General Plant Pathology, 72, 6–15.

[asj13141-bib-0032] Surai, P. F. (2002). Natural antioxidants in avian nutrition and reproduction. Nottingham, UK: Nottingham University Press.

[asj13141-bib-0033] Valko, M. , Leibfritz, D. , Moncol, J. , Cronin, M. T. , Mazur, M. , & Telser, J. (2007). Free radicals and antioxidants in normal physiological functions and human disease. The International Journal of Biochemistry and Cell Biology, 39, 44–84. 16978905 10.1016/j.biocel.2006.07.001

[asj13141-bib-0034] Whitehead, C. C. , & Keller, T. (2003). An update on ascorbic acid in poultry. World Poultry Science Journal, 59, 161–184.

[asj13141-bib-0035] Wilson, B. R. , Pearson, A. M. , & Shorland, F. B. (1976). Effect of total lipids and phospholipids on warmed‐over flavor in red and white muscle from several species as measured by thiobarbituric acid analysis. Journal of Agriculture and Food Chemistry, 24, 7–11.

[asj13141-bib-0036] Zeferino, C. P. , Komiyama, C. M. , Pelícia, V. C. , Fascina, V. B. , Aoyagi, M. M. , Coutinho, L. L. , … Moura, A. S. A. M. T. (2016). Carcass and meat quality traits of chickens fed diets concurrently supplemented with vitamins C and E under constant heat stress. Animal, 10, 163–171. 26677935 10.1017/S1751731115001998

[asj13141-bib-0037] Zhang, Z. Y. , Jia, G. Q. , Zuo, J. J. , Zhang, Y. , Lei, J. , Ren, L. , & Feng, D. Y. (2012). Effects of constant and cyclic heat stress on muscle metabolism and meat quality of broiler breast fillet and thigh meat. Poultry Science, 91, 2931–2937. 10.3382/ps.2012-02255 23091152

